# Commentary: Feedback stabilizes propagation of synchronous spiking in cortical neural networks

**DOI:** 10.3389/fncom.2015.00071

**Published:** 2015-06-10

**Authors:** Enric Claverol-Tinturé, Guenter Gross

**Affiliations:** ^1^Catalonia Foundation for Science and InnovationBarcelona, Spain; ^2^Center for Network Neuroscience, University of North TexasDenton, TX, USA

**Keywords:** spiking neuronal networks, feedback, timing, brain evolution, oscillations, synchronization

Recently published computational work (Moldakarimov et al., 2015) investigated local network feedback as a physiologically plausible strategy for the preservation of precise spike timing in propagating volleys.

Since the timing of spikes contributes to conveying and processing information across species, information modalities and brain areas (Singer, 1999; Jermakowicz and Casagrande, 2007; Harvey et al., 2013; Zuo et al., 2015), it is reasonable to expect that nature has developed strategies to retain, when needed, narrow spike-time spreads in population activity as it propagates along multi-synaptic paths.

Indeed, it has been demonstrated computationally that weak stimuli and the resulting sparse volleys are at risk of propagation failure and desynchronization as activity traverses multiple neuronal layers (Diesmann et al., 1999; Vogels and Abbott, 2005). Moldakarimov and colleagues show that feedback can do the trick and preserve spike timing even in weak propagating volleys. They first simulated a 5-layer purely feedforward network with Hodgkin-Huxley conductance cell models and observed how weak stimulation of layer 1 did not succeed in reaching layer 5 with similar intensity and low jitter. However, adding feedback connections from layer N to layer N-1, targeting both excitatory and inhibitory cells, succeeds in propagating sparse activity with narrow timing spreads.

These results merit consideration from an evolutionary perspective in the context of the wider problem of brain scalability. Throughout evolution brain mass increased and neuronal aggregates reorganized (Clark et al., 2001; Herculano-Houzel et al., 2007, 2014). Remarkably, many anatomically and functionally distinctive modules in the mammalian brain have been preserved and enlarged by evolution, partially substantiating the hypothesized scalability of brain's architectural plan (Clark et al., 2001). Yet much remains to be understood regarding the computational advantages of brain size, the limits of scalability and its compatibility with the biophysical constraints imposed by neuronal tissue.

One such biophysical constraint stems from the finite conduction velocity of axons (Swadlow et al., 1978). Bigger brains with longer propagation paths and increased end-to-end delays could hamper the synchronization of activity among distant areas. Instead, oscillatory dynamics with isochronous discharges do occur over widely distributed areas, irrespective of brain size (Roelfsema et al., 1997; Varela et al., 2001; Uhlhaas et al., 2009).

Various compensatory mechanisms have been proposed to counter delays and support scalability. Buzsáky and colleagues hypothesized that a subpopulation of heavily myelinated axons with larger than average diameters might be eventually discovered where and when faster conduction velocities and moderate time lags are needed (Buzsáki et al., 2013). An alternative, dynamic relaying, proposes that intermediate populations linked by recurrent connections to distant areas can induce their synchronization despite relatively large time lags (Vicente et al., 2008; Barardi et al., 2014; Gollo et al., 2014).

Prioritizing first spike timing to spike rates as information carrier over afferent tracks could also confer scalability. Consider for example the human visual system, capable of categorizing images within an astonishing 100 ms, as demonstrated by local field potentials in the inferior occipital gyrus recorded from epileptic patients undergoing surgery (Liu et al., 2009; Tang et al., 2014).

It stands to reason that the enlargement of the brain throughout evolution likely resulted in the flow of visual information from the retina necessarily traveling longer distances to reach high-level cortical areas. How could the visual system be scalable and robust against growing delays? Perhaps favoring first spike timing as information carrier played a role (Thorpe et al., 1996; Gutig et al., 2013). Single spike processing could be fast and more resilient against brain-size related delays than spike rate computation because the latter requires the observation of relatively long epochs of spike trains. With first spike computation fast image categorization could then be retained despite increasing brain size and scalability and robustness against delays might ensue.

It is against this background of ongoing research on the limits of brain scalability related to brain size that the work by Moldakarimov and colleagues can be seen as relevant to the broader endeavor of understanding brain evolution. They explore feedback as a compensatory mechanism that counters undesired intra-volley desynchronisation which could arise as new tissue and synaptic layers were added by evolution. Their work adds to very relevant research on synfire chains (Abeles, 1982; Jahnke et al., 2013) often approaching also the problem of desynchronization of volleys.

In the coming years it is likely that the emerging connectome will shed light on the subject elucidating further compensatory mechanisms developed by evolution. A myriad of techniques including Diffusion Tensor Imaging (DTI), automated 3D reconstruction from optical and electron microscopy data, fMRI, EEG, MEG, and others, are increasingly supporting a concerted effort for systematic whole-brain anatomical and functional mapping of brain connectivity (Devor et al., 2013; Van Essen, 2013; Hale, 2014; Mitra, 2014; Eberle et al., 2015). Connections undergoing evolution-driven elongation, potentially increased time lags, as well as structures with multilayer cytoarchitectures at risk of desynchronizing weak volleys as new layers are added, will be systematically identified. The hypothesized compensatory strategies, being increased myelination and thicker axons, local feedback connections, dynamic relay architectures or others will be eventually confirmed or refuted.

Computational neuroscientists are uniquely positioned to tackle the grand challenge of understanding brain evolution. Simulations can efficiently validate the functional advantages of presumed evolutionary events and the correct sequence in which these took place. The work of Moldakarimov and colleagues contributes toward solving this puzzle and motivates further research.

## Conflict of interest statement

The authors declare that the research was conducted in the absence of any commercial or financial relationships that could be construed as a potential conflict of interest.
